# Teaching trainers to incorporate evidence-based medicine (EBM) teaching in clinical practice: the EU-EBM project

**DOI:** 10.1186/1472-6920-9-59

**Published:** 2009-09-10

**Authors:** Shakila Thangaratinam, Gemma Barnfield, Susanne Weinbrenner, Berit Meyerrose, Theodoros N Arvanitis, Andrea R Horvath, Gianni Zanrei, Regina Kunz, Katja Suter, Jacek Walczak, Anna Kaleta, Katrien Oude Rengerink, Harry Gee, Ben WJ Mol, Khalid S Khan

**Affiliations:** 1 Birmingham Women's NHS Foundation Trust, Metchley Park Road, Edgbaston, Birmingham B15 2TG, UK; 2 University of Birmingham, Edgbaston, Birmingham B15 2TG, UK; 3 Agency for Quality in Medicine, Wegleystrasse 3, 10623 Berlin, Germany; 4 TUDOR, University of Szeged, Albert Szent-Gyorgyi Medical and Pharmacological Centre, Somogyi Bela ter 1, Szeged, H-6725, Hungary; 5 Università Cattolica del Sacro Cuore, Via Emilia Parmense 84, 29100 Piacenza, Italy; 6 Basel Institute for Clinical Epidemiology, Hebelstrasse 10, CH 4031 Basel, Switzerland; 7 CASPolska, 30-347 Krakow, ul. Wadowicka 3, Poland; 8 Academic Medical Center, University of Amsterdam, Department of Obstetrics and Gynaecology, Meibergdreef 9, 1105 AZ Amsterdam, the Netherlands

## Abstract

**Background:**

Evidence based medicine (EBM) is considered an integral part of medical training, but integration of teaching various EBM steps in everyday clinical practice is uncommon. Currently EBM is predominantly taught through theoretical courses, workshops and e-learning. However, clinical teachers lack confidence in teaching EBM in workplace and are often unsure of the existing opportunities for teaching EBM in the clinical setting. There is a need for continuing professional development (CPD) courses that train clinical trainers to teach EBM through on-the-job training by demonstration of applied EBM real time in clinical practice. We developed such a course to encourage clinically relevant teaching of EBM in post-graduate education in various clinical environments.

**Methods:**

We devised an e-learning course targeting trainers with EBM knowledge to impart educational methods needed to teach application of EBM teaching in commonly used clinical settings. The curriculum development group comprised experienced EBM teachers, clinical epidemiologists, clinicians and educationalists from institutions in seven European countries. The e-learning sessions were designed to allow participants (teachers) to undertake the course in the workplace during short breaks within clinical activities. An independent European steering committee provided input into the process.

**Results:**

The curriculum defined specific learning objectives for teaching EBM by exploiting educational opportunities in six different clinical settings. The e-modules incorporated video clips that demonstrate practical and effective methods of EBM teaching in everyday clinical practice. The course encouraged focussed teaching activities embedded within a trainer's personal learning plan and documentation in a CPD portfolio for reflection.

**Conclusion:**

This curriculum will help senior clinicians to identify and make the best use of available opportunities in everyday practice in clinical situations to teach various steps of EBM and demonstrate their applicability to clinical practice. Once fully implemented, the ultimate outcome of this pilot project will be a European qualification in teaching EBM, which will be used by doctors, hospitals, professional bodies responsible for postgraduate qualifications and continuing medical education.

## Background

Evidence-based medicine (EBM) is considered to be the most ethical way to practice medicine as it integrates research into clinical practice. It has evolved into a powerful tool for well informed decision-making within the clinician's daily practice. The challenge is to teach it practically in a clinical setting [1-4]. Unless trainees practice EBM in everyday clinical work and observe their teachers practice EBM it will be difficult for EBM to achieve the status of core competency in medicine. To develop effective on-the-job training requires clinical teachers to develop the confidence to explicitly demonstrate to trainees how their daily decisions about healthcare are based on the best available, current, valid and relevant evidence [3]. For this, they need to move their trainees forward from standalone courses, which may be effective in improving EBM knowledge, to clinically integrate work-based teaching and learning that can bring about changes in skills, attitudes and behaviour [5,6]. How can senior clinicians develop competence in teaching integration of good quality research evidence into clinical practice?

Through our EU EBM Unity project we had successfully devised a clinically integrated EBM e-learning course targeting the *learners *of EBM [7,8]. The publication of this project was "Highly Accessed" at Bio Med Central [7]. Often the knowledge and skills attained by the EBM learners from courses like the above fell into decline due to lack of opportunities to continue EBM practice in their clinical environment. During the development of our EBM course for learners, we became aware of the need to equip trainers with practical teaching skills to teach trainees the use of EBM in various clinical activities. There is a need to develop continuing professional development (CPD) courses aimed at clinical teachers based on the sound principles of effective continuing education. No such courses currently exist for teaching the trainers effective methods for teaching and demonstrating integration of EBM to their trainees in a clinical setting. This background led us to develop a clinically integrated course for the *teachers *of EBM who supervise postgraduate medical training. This paper describes the process of curriculum development along with its current results.

## Methods

Funded by the European Union (EU) through the Lifelong learning programme 2007 Leonardo da Vinci Transfer of innovation pilot project, we designed an e-learning curriculum for CPD that would encourage participants (trainers) to learn practical and effective teaching methods for tutoring application of EBM in various clinical settings. The EU EBM TTT partnership involves a collaboration of 10 partners within Europe and contributes to harmonisation of EBM learning and teaching across the European healthcare sector .

We developed the curriculum for teachers using sound educational principles for effective adult learning allowing a maximum of flexibility and relevance to clinical practice [9-11]. A curriculum committee consisting of experts in the field of EBM, clinical epidemiologists, clinicians and educationalists from the participating countries contributed to the work. Our methodology for the curriculum design included identification of learning needs in each partner country, formulation of the aims, objectives and learning outcomes, development and organisation of the content of the curriculum, development of the teaching methods, definition of the educational strategy and educational environment, and delineation of an assessment strategy.

In addition to our working group, an independent European Steering Committee provided input into the content, educational approach, applicability and sustainability, giving advice at critical stages in the curriculum development and thereby assured external validation and triangulation of the syllabus prepared.

## Results

### Identification of learning needs of EBM trainers

To begin with we identified specific educational opportunities in various clinical settings that can be exploited by trainers to teach application of EBM. We took into account the variation in clinical settings and teaching opportunities in different countries along with the variation in the level of EBM knowledge amongst the trainers and trainees. This was done by an in-depth discussion among the project partners who had a good overview of the ongoing activities in their countries, with input from the external steering committee. We performed a survey to identify the commonly perceived obstacles for teaching EBM in clinical practice. The feedback from trainers in our earlier work showed that there was a considerable demand for up-skilling trainers [12]. The above process helped to define feasible educational goals for the documented learning needs of clinical trainers.

### Formulation of objectives and learning outcomes of teaching the EBM trainers course

The aim of the e-course was to provide trainers with practical tips and methods by which EBM can be taught during a range of clinical activities. The course had a large e-learning component providing practical demonstration of techniques to teach EBM to clinical trainees [13]. E-learning sessions were designed to allow learning in the workplace during short breaks within clinical activities, with the option to interrupt and restart learning flexibly. The learning objectives and outcomes were to help participants identify their trainees' knowledge gaps related to current clinical practice and to use these to initiate EBM teaching (Appendix). This would allow them to teach the trainees to follow the key steps of EBM in practice. They would generate structured questions in an appropriate format in any clinical setting e.g. during ward round, outpatient clinics, case discussion meeting, etc. Trainers should be able to guide the trainees to search for relevant literature, aiming for and identifying systematic reviews wherever possible by helping them choose the relevant search terms and directing them towards appropriate databases with input from a medical librarian if possible [14]. They should be able to teach the trainee to assess the quality (validity) of systematic reviews and the primary research included within them, emphasising that rather than being a statistical exercise it is actually an exercise towards bridging the gap between research and practice [15]. They should be able to demonstrate the applicability of the research findings to current clinical practice. Finally, they should encourage the trainees to evaluate and eventually adapt current practice in the light of newer evidences through clinically relevant activities like audit or quality assurance [16]. These key issues guided the development of the curriculum in six modules. Each module exploited learning and teaching opportunity presented at various clinical settings and provided practical tip to teach the key steps of EBM.

### Content of modules

The curriculum was divided into six modules each of which presented approaches to exploit a learning opportunity in formal and informal clinical settings for teaching application of the five steps of EBM (Table 1). These clinical settings were:

**Table 1:** Learning opportunities for teaching evidence based medicine (EBM) in clinical practice

**EBM Steps**					
	
**Learning Opportunities**	**Formulating Questions**	**Searching for Evidence**	**Critical Appraisal**	**Integrating Evidence with Clinical scenario**	**Bringing Change to Practice**
Ward Round	+++	(+)	+	+++	+
Journal Club	+	++	+++	+	+
Clinical Teaching And Assessment	++	(+)	++	+++	+
Outpatients Clinic	+++	(+)	++	+++	+
Formal Clinical Meeting	+++	(+)	+	++	+
Audit	++	++	++	+	+++

Each clinical setting offers varying opportunities to teach application of the 5 EBM steps.

(+) Opportunity may arise + Minimal opportunity

++ Moderate opportunity +++ Maximum opportunity

1. Ward rounds: These are usually undertaken by a senior clinician with trainees, nurses and if feasible clinical librarian

2. Journal club: It is a regular educational activity where the clinician, often a trainee critically appraises a published piece of evidence obtained by systematic search of literature to answer a clinical question

3. Formal clinical meetings: Any meeting attended by the trainer or trainee from one or more disciplines where the management of individual cases are discussed

4. Outpatient clinic: The management of patients on an outpatient basis by the trainer and trainee

5. Audit meeting: It is a formal meeting usually held in a hospital where current clinical practice is compared to predetermined standards of care

6. Formal clinical assessment: It is usually a one to one meeting between the trainer and the trainee where the trainer assesses the clinical knowledge and skills of the trainee using validated assessment tools

**Figure 1:**
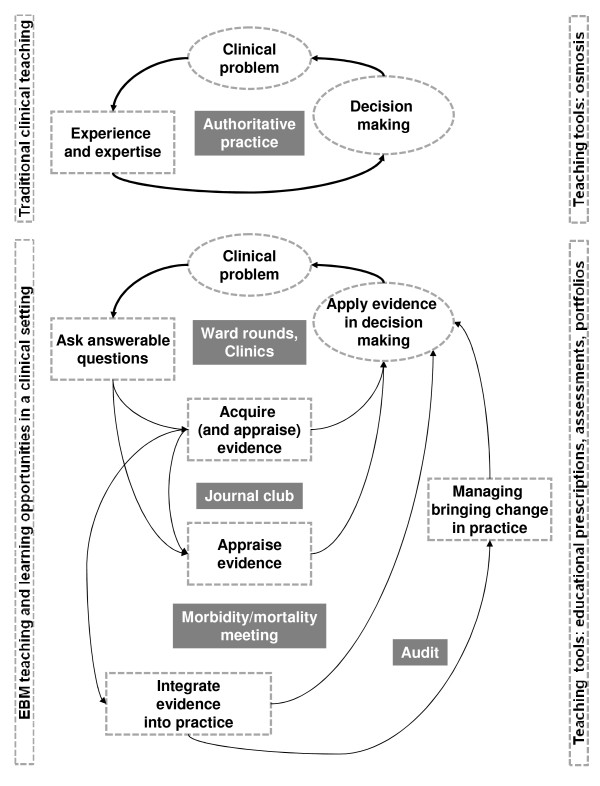
**Clinical tools to teach various steps of EBM in different clinical settings**.

Each module outlined the basic prerequisite EBM competencies to be acquired by participants to fulfil the qualification as EBM teachers. The clinical setting was described taking into account the variation in practice and settings between healthcare systems in different countries. Where appropriate we have provided guidance for adaptation of the teaching methods according to the existing resources and opportunities. Practical advice was provided on facilitating teaching of the various EBM steps with videos demonstrating EBM teaching in action (Fig 1) [13]. Our e-learning package from our previous project has been shown perform as well as face to face teaching [17,18].

Teaching opportunities during clinical encounters were taught through two modules focussing on ward rounds and outpatient clinics [14]. These two clinical settings are the commonest available opportunities for interaction between the trainer and trainee. The first EBM step, question formulation, can easily be taught here. The module helps the teacher to expose knowledge gaps in the trainee by asking questions relevant to the patient seen in the clinic or ward and framing them as focussed question using the PICO format. It guides the trainer how to prescribe an educational prescription which can be quickly handed to a trainee for them to follow it up when an opportunity arises during their busy schedule [19]. In institutions where there are facilities to perform immediate searches, a clinical librarian or another colleague can demonstrate the ways in which trainees can search in appropriate databases using relevant search terms to obtain evidence.

Opportunities to teach EBM during clinical meetings were covered in three modules using Journal club, Morbidity and Mortality meetings, and Audit presentations [16,20,21]. These occasions engage trainees in communal EBM activity. These meetings offer the trainee the chance to prepare in advance before the event. The modules illustrate with the help of video demonstration and slides, the role of the teacher in providing the trainee with information and material on the literature searching and critical appraisal steps of EBM. Journal clubs are ideal opportunities to teach in depth critical appraisal of papers presented with help from easy to use software for EBM calculations [20,21]. At morbidity and mortality meetings, in addition to obtaining and appraising the relevant evidence, the ways in which the applicability of that evidence to the care of the individual patient can also be illustrated. The use of Audit meetings in teaching the final step in EBM, bringing change to clinical practice, can be demonstrated [16].

Clinical assessment of trainees are ideal opportunities for the teacher to provide formative or summative assessment of EBM competence and for giving feedback on their performance [13,22]. These assessments are carried using different tools in different countries. The module also provided examples of assessment tools used in different European countries.

### Administration of the course and learning method

**Figure 2:**
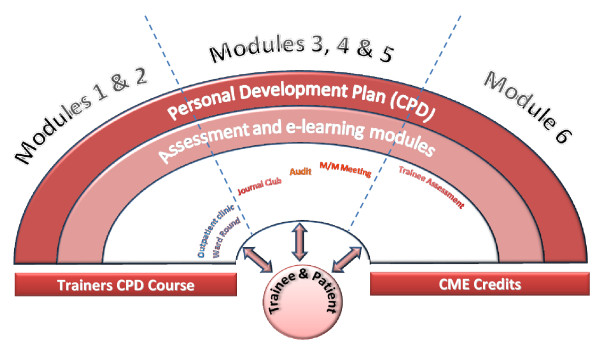
**An overview of teaching and learning activities in the EU EBM Training the Trainers (TTT) course: Graphical representation of the EU-EBM TTT curriculum**.

The 6 module course had been developed for independent study. Participants will be supervising senior doctors who act as moderators in the learning process of their trainees. They will normally be competent practitioners who will employ the tips from the e-learning modules to identify teaching opportunities encountered in daily clinical care for patients and by directing appropriate use of learning resources in a clinical setting. The course can be accessed via the Internet from the website , USB or CD-ROM. In addition to the above methods, we aim to provide access through video podcasts and social websites like Face book. Furthermore it will enable busy clinicians to access the course both inside and outside the hospital, target a wider audience and promote dissemination. The e-modules integrate a combination of teaching methods (Fig 2). They are developed to achieve self-directed, independent e-learning. At the start of the course the participants are provided with the overall aims and objectives of the curriculum, the number and title of modules each of which represent a clinical setting, an outline of the teaching, learning and assessment strategy, any relevant links or methodological papers and the time taken to complete each module. The course is developed to enable the participants to complete the modules in 15 to 20 minutes. The e-learning package consists of slides and written scripts; a talking head which covers the content of the scripts and guides the lecture; play, pause and skip options; and hyperlinks to main sections within the sessions. There is a short 3 minutes video clip of the practical demonstration of interaction between the trainer and trainee in the different clinical settings. Sessions can be accessed multiple times if necessary. The participants will apply the knowledge acquired from modules into daily teaching of their trainees and will document their progress. At the end of the modules the participant will complete an assessment using multiple choice questions that includes the content of the course. The assignments will contribute to their personal development plan.

### Assessment method

Our systematic review of existing assessment tools has not identified specific assessment tools for evaluating effectiveness of teaching the teachers courses in EBM[23] We therefore developed and validated a questionnaire to assess the participants' performance before and after completion of the course. They will be assessed on their knowledge, skills and attitude using specific questions in a multiple choice format. The course has been piloted in 5 EU countries involving 60 participants. During the piloting we have collected feedback from the participants on the content of course material, ease of access, clarity of information provided using semi structured qualitative interviews. We have evaluated the technology acceptance using a validated Technology Acceptance Model (TAM) questionnaire. The results of these will enable further improvement of the course to suit the specific needs of participants.

## Discussion

Through this EU project we have established an e-learning course targeting EBM teachers with emphasis on its piloting and promotion across the European healthcare sector and beyond.

It is well known that effective learning occurs when learners link theory with practice. This implies that learners are able to make connections between knowledge gained in one scenario and apply it to another [24]. They are able to achieve this by identifying and exploiting the educational opportunities available in daily clinical practice learnt through the course to teach integration of EBM. This curriculum will help senior clinicians to make the best use of available opportunities in everyday clinical situations to teach about the various steps of EBM and their application to practice, using the principles of adult learning. It will contribute to their professional development and will improve their educational environment.

The e-curriculum for trainers satisfies the CRISIS criteria for successful CPD [10]. It is developed in such a way that it is *Convenient *(C) for the trainer to learn integration of EBM teaching in clinical practice by working through the modules in a setting and time of their choice. To our knowledge at the moment such a curriculum aimed at this target population does not exist. By showing the teaching opportunities in various clinical settings encountered by any healthcare professional in day-to-day practice, it is highly *Relevant *(R) to this group of individuals. The curriculum is *Individualised *(I) with flexibility to apply the principles of teaching in various clinical settings, medical disciplines and countries. Where possible every effort has been made to ensure that the variations in healthcare system across the EU countries are taken into account during the curriculum development. At the end of modules, there is the prospect of *Self-assessment *(S) of the participants to evaluate their knowledge and competency in teaching EBM. The assessment tools have been developed and validated and has been piloted in the partner countries. Use of short segments of slides combined with video clip recordings of the actual EBM teaching in various clinical set up is aimed at arousing the *Interest *(I) of the learner. By highlighting the differences in clinical practice, teaching techniques, available resources and educational culture we have attempted to *Speculate *(S) on difficult and controversial areas of the course. The entire e-curriculum is devised in such a way that the learning process is *Systematic *(S) with specific aims, learning objectives, content and summary for each module.

The use of the curriculum may be limited by the differences in clinical settings, EBM knowledge, resources and attitudes in various institutions and countries. The examples provided in the modules are limited to articles on therapy. With increasing use of the modules in various countries will help in further development with necessary adaptations as required.

The goal of this project is to help clinical trainers identify opportunities to teach trainees integration of EBM, provide them with tips to perform this in various clinical settings[13] and to raise awareness about this curriculum for trainers throughout Europe and ultimately incorporate it in mainstream curriculum. The dissemination systems in place such as the website, presentations on national conferences and workshops, word of mouth and publications in national language will allow access, discussion and dissemination of the results. The project partners are exploring ways to involve their national networks of institutions that might benefit from the process.

Currently in this project we have covered ways to teach EBM in six clinical settings. Furthermore to complement our previous project on EBM curriculum, we have provided examples using systematic reviews of therapy. We are translating the course in six languages. In the future, we plan to increase the number of modules to cover other clinical settings not currently included, provide examples using diagnostic and prognostic studies and make the curriculum relevant to healthcare professionals other than doctors.

## Conclusion

The EU EBM Training the Trainers curriculum will help senior clinicians to identify and make the best use of available opportunities in everyday practice in clinical situations to teach various steps of EBM and demonstrate their applicability to clinical practice. Complete application of this pilot project aimed at teachers of EBM will be a European qualification in teaching EBM, which will be used by senior doctors, hospitals and professional bodies responsible for postgraduate qualifications and continuing medical education. The EU EBM Training the Trainers project will lead the way in bringing about teaching EBM closer to everyday clinical practice with the prospect of improving patient care.

## Competing interests

The authors declare that they have no competing interests.

## Authors' contributions

ST, KSK and TNA drafted the first version of the manuscript. GZ developed the website. RK, BWM, KO, KS, JW, AK, SW, BM, GB, HG and ARH revised the manuscript and approved final submission for publication. All authors are members of the EU-EBM and were involved in the design, development and implementation of the course described.

## Appendix: An overview of e-EBM Training the Trainers continuing professional development (CPD) course

### Aim

To provide guidance on practical and effective methods to teach clinically integrated evidence based medicine (EBM) in various clinical settings

### Target participants

Senior health professionals in a clinical setting with responsibility for teaching and training.

### Learning objectives

Upon completion of the course participants should be confidently able to identify and use learning opportunities in a clinical setting to provide on-the-job training to trainees by

- exposing knowledge gaps in the trainees that leads to construction of structured question

- seeking information from trainees on how they will track the best evidence to answer the question

- developing confidence in number crunching to interpret the results

- effectively using various clinical opportunities to teach critical appraisal of literature

- demonstrating how clinical judgement is used to determine the extent to which research evidence can be applied for individual patient care

- enabling trainees to present and discuss in formal clinical meetings by applying the steps of EBM

### Learning/teaching methods

Participants to pursue independent study by using the following e-learning modules to obtain CPD or continuing medical education (CME) credits

E-learning modules with demonstration videos

• Module 1: Ward Rounds

• Module 2: Journal Club

• Module 3: Clinical assessment

• Module 4: Outpatients Clinic

• Module 5: Formal Clinical Meeting

• Module 6: Audit

### Assessment

Questionnaire and log of teaching activities in a personal learning plan

## Pre-publication history

The pre-publication history for this paper can be accessed here:


